# Intracellular Delivery of Molecular Cargo Using Cell-Penetrating Peptides and the Combination Strategies

**DOI:** 10.3390/ijms160819518

**Published:** 2015-08-18

**Authors:** Hua Li, Tung Yu Tsui, Wenxue Ma

**Affiliations:** 1 Department of Basic Medical Science, Huzhou University School of Medicine, Huzhou 313000, China; E-Mail: lihua@hutc.zj.cn; 2 Department of General, Visceral and Thoracic Surgery, University Medical Center Hamburg-Eppendorf, Martinistr. 52, 20246 Hamburg, Germany; 3 Moores Cancer Center, University of California San Diego, La Jolla, CA 92093-0820, USA

**Keywords:** cell-penetrating peptides (CPPs), TAT peptide, liposome, nanoparticles, combination delivery, calcium

## Abstract

Cell-penetrating peptides (CPPs) can cross cellular membranes in a non-toxic fashion, improving the intracellular delivery of various molecular cargos such as nanoparticles, small molecules and plasmid DNA. Because CPPs provide a safe, efficient, and non-invasive mode of transport for various cargos into cells, they have been developed as vectors for the delivery of genetic and biologic products in recent years. Most common CPPs are positively charged peptides. While delivering negatively charged molecules (e.g., nucleic acids) to target cells, the internalization efficiency of CPPs is reduced and inhibited because the cationic charges on the CPPs are neutralized through the covering of CPPs by cargos on the structure. Even under these circumstances, the CPPs can still be non-covalently complexed with the negatively charged molecules. To address this issue, combination strategies of CPPs with other typical carriers provide a promising and novel delivery system. This review summarizes the latest research work in using CPPs combined with molecular cargos including liposomes, polymers, cationic peptides, nanoparticles, adeno-associated virus (AAV) and calcium for the delivery of genetic products, especially for small interfering RNA (siRNA). This combination strategy remedies the reduced internalization efficiency caused by neutralization.

## 1. Introduction

Cell-penetrating peptides (CPPs) are peptides composed of between four to 30 amino acids that are usually rich in arginine and lysine residues, and are characterized by their unique internalization properties. CPPs include protein transduction domain (PTD) [1,2], trojan peptides [3], arginine-rich peptides [4], and vectocell peptides, which are also known as Diatos Peptide Vectors (DPV) [5]. In general, the entry mechanisms of CPPs include (1) direct penetration (energy-independent pathway) into the cell membrane; (2) endocytosis-mediated entry (energy dependent pathway) that the plasma membrane folds inward to bring molecular cargos into cells; and (3) translocation through the formation of a transitory inverted micelles, which are aggregates of colloidal surfactants where the polar groups are concentrated in the interior and the lipophilic groups extend outward into the solvent. Although significant efforts have been made to elucidate the exact translocation mechanisms of the CPPs [6,7,8], various factors, such as the conjugation of molecular cargos and CPPs, cell types, concentration, and the types and structure of CPPs, may affect the internalization of CPPs [9,10,11,12]. The cellular uptake of therapeutic cargos becomes a major obstacle in the clinical setting and biological fields due to the lipid bilayer of the cell membrane. Since the first cell-penetrating protein was found and derived from immunodeficiency virus (HIV) with a high cellular uptake efficiency [13], numerous CPPs have been gradually discovered and utilized as carriers in the past decades. Some of the CPPs are summarized in Table 1, and other CPPs can be found in the database [14]. Among the CPPs, three main penetration mechanisms are as follow: (1) direct penetration [7], a reaction that is more favorable at high concentrations of CPPs—it occurs via an energy-independent pathway by a direct electrostatic interaction of positively charged CPPs with negatively charged phospholipids or heparin sulfate of membrane components; (2) Endocytosis [15,16], an energy-dependent process of cellular ingestion by which cells adsorb the external materials; and (3) Macropinocytosis, a form of endocytosis that accompanies cell surface ruffling [17]. Furthermore, a recent research reported that calcium influx is also involved in the process of intracellular delivery of CPPs [18].

**Table 1:** Representative CPPs.

Name	Sequence	References
Poly-R peptides	R*x* *	[19,20,21,22]
TAT (47–57)	YGRKKRRQRRR	[23]
TAT (49–60)	RKKRRQRRRPPQ	[21]
PTD4	YARAAARQARA	[24]
PTD5	RRQRRTSKLMKRGG	[25]
TP10	AGYLLGKINLKALAALAKKIL	[26]
M918	MVTVLFRRLRIRRACGPPRVRV	[27]
pAntp (43–58) [Penetratin]	RQIKIWFQNRRMKWKK	[21]
KNO	KQINNWFINQRKRHWK	[28]
Hph-1	YARVRRRGPRR	[29]
HIV-1 Rev (34–50) ANP (43–58) [Antennapedia]	TRQARRNRRRRWRERQR-GC RQIKIWFQNRRMKWKK-GC-CO	[30]
POD	GGG [ARKKAAKA] 4	[31]
S413-PV	ALWKTLLKKVLKAPKKKRKV	[32]
S41	CVQWSLLRGYQPC	[33]
pVEC	LLIILRRRIRKQAHAHSK	[34]

* *x* = 4, 8, 10, 11.

The CPPs can transfer various molecule cargoes including peptides [35], proteins [36,37,38], plasmid DNA [39,40], and nanoparticles [39,41,42]. Recently, Dr. Singh and his colleagues reported that CPPs tethered bi-ligand liposomes and short-chain CPPs are capable of crossing the blood brain barrier (BBB) [2,43,44]. The frequency and kinetics of CPPs transfection varies between different cell types. Furuhata *et al.* [19] showed the Arg4 was the most efficient among the (Arg)n (*n* = 4, 6, 8, 10) when internalized in HeLa cells, and the efficiency decreased depending on the length of oligo-Arg. In living neurons, the R11 peptide showed the highest transduction efficiency when compared with other R*x* peptides such as R5, R7 and R9 [45]. Trans-Activator of Transcription (TAT), an arginine-rich CPP, was more efficient than R9 in living neurons; however, the conclusion was the contrary in rat alveolar epithelial cells [46].

**Figure 1:**
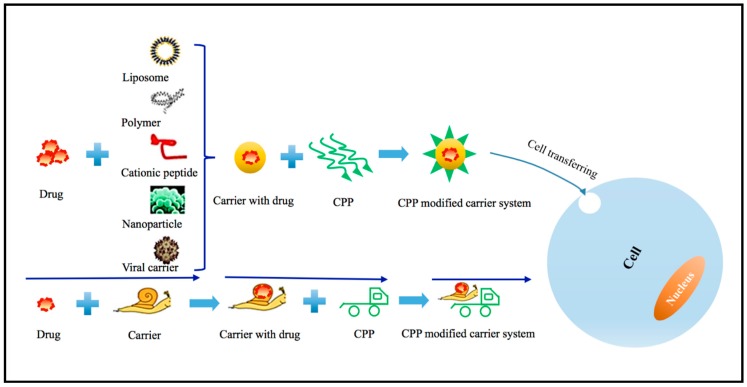
Enhanced drug delivery system by the combination of CPPs with other carriers. Drugs (far left) can be incorporated into the typical carrier systems, which can be modified with CPPs. Under this circumstance, the CPPs-modified drug carrier system not only dramatically enhances the intracellular delivery efficiency, but also improves the endosomal escape through the alteration of its biodistribution.

Although CPPs have been notably developed to mediate the intracellular delivery of the nucleic acids with negative charge [1,47], the major drawback is the free high cationic CPPs that could bind to negatively charged nucleic acids molecules. This binding results in the aggregation of CPPs-nucleic acids and cytotoxicity. Furthermore, the ability of cell penetration will be inhibited or lost due to the neutralization of the CPPs by negatively charged molecules [48,49]. To overcome these difficulties and improve the delivery efficiency of genetic products (particularly siRNA), other carrier systems can be developed via the conjugation with CPPs (Figure 1).

## 2. CPPs-Modified Liposome

Once hydrophilic lipid and hydrophobic lipid chain at opposite ends, they form liposomes when exposed to water at appropriate conditions. According to the size of lamellarity and formation, the liposome is classified into multilamellar vesicles (500 to 5000 nm), small unilamellar vesicles (around 100 nm), and large unilamellar vesicles (200 to 800 nm). During the liposome formation, the water-soluble drug molecules will also be packed into the inner water space of liposome [50]; this specific geometry protects the active agent from the destructive tendencies of the external environment. In addition, liposomes are capable of crossing membranes to deliver its contents into cells and cell compartments. However, while it can transfer diverse active components to the target cells, the low transfer efficiency limits its further application in the clinical setting.

CPPs-modified liposome can improve the efficiency of cell penetration [51]. The efficiency of liposome surface modified with Antennapedia (Antp, aa 43–58) or TAT (47–57) is 15 to 25 folds higher than that of non-modified liposome [52]. Both CPPs-modified [53] and octaarginine-coated [53,54] liposomes resulted in an increased rate of liposome uptake. The cell penetration efficiency of TAT-liposome is dramatically improved by 1000 times as opposed to the TAT only [9]. Consequently, CPPs-modified polyarginine8 (R8)-liposome has been used for siRNA delivery into lung cancer cell lines with remarkably higher transfection efficiency. The siRNA degradation in the blood serum is significantly inhibited, and showed a lower non-specific toxicity [55]. Although there is no significant difference between cellular uptake of R8-liposome and the conventional cationic liposome [56], the CPPs-modified liposome can improve the capacity of the CPPs endosomal escape from the liposome surface as well as the cargo delivery efficiency of the liposome. Thus, there are two important parameters in the CPPs-modified liposome. One is the threshold amount of CPPs on the liposome surface, and the other is the type of CPPs: (1) peptides derived from proteins; (2) chimeric peptides that are formed by the fusion of two natural sequences; and (3) synthetic CPPs, which are rationally designed sequences usually based on structure–activity studies.

**Figure 2:**
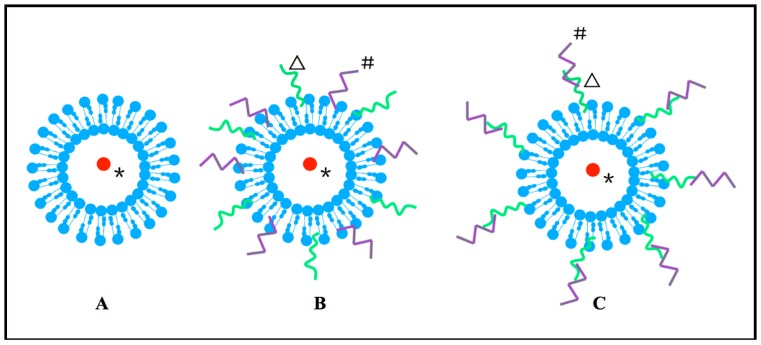
CPPs-modified liposome. (**A**) The original liposome; (**B**) The CPPs conjugated directly to the surface of the liposome; (**C**) The CPPs modified to the surface of PEG first, and then conjugated to the liposome. The blue circle in the figure panel typifies liposome, # represents the CPPs, Δ stands for other modified cargo (e.g., PEG) and * symbolizes the transferred cargo.

To circumvent the fast elimination of liposome in blood, surface-modification methods, such as the PEG-modified strategy, are used to achieve long circulation of liposomes *in vivo.* The CPPs TAT peptide can be attached to the liposome surface via the liposomal NGPE (TAT-LIP-NGPE) or the PEG spacer by coupling with pNP-PEG-PE (LIP-pNP-PEG-TAT) [57], as shown in Figure 2. While the long PEG (PEG 5000) chains attached to the liposome might interfere with the TAT peptide in cell surface interactions and decrease the effectiveness, the TAT peptide-modified liposomes, on the other hand, have no obstacles for this interaction. PEG 2000 [58] or PEG 3000 [57] and TAT-modified liposomes can also deliver target drugs efficiently into the cells. Although TAT, as a cellular uptake enhancer, can be hidden by long chain PEG, this problem can be avoided by degrading the latter before TAT penetrates the cell membrane, thus protecting TAT from degradation [59].

The key barrier for the treatment of central nervous system (CNS) diseases is the BBB penetration. CPPs exhibited the promising advantage of freely trafficking across the BBB. With the aim of increasing the amount of drug that could reach the CNS system, CPPs-conjugated liposomes were produced and showed higher brain delivery efficiency [60,61]. Similarly to CPPs, TAT-conjugated PEGylated magnetic polymeric liposomes (MPLs), TAT-PEG-MPLs are consumed more in the injured spinal cord and inside the neuron when compared to non-TAT-modified MPLs [62].

## 3. Combination of CPPs with Polymer

Polymer is a popular drug-carrying material that makes nanoparticles and micelles, which can deliver siRNA, DNA, protein, as well as hydrophilic and hydrophobic cargos, and the cationic polymer is a new type of siRNA delivery vector. Currently, multiple substructures have been established in one carrier, and each of them has a special function in the siRNA delivery process. For example, Poly-l-Lysine (PLL), a synthetic chiral polymer, acts as the positive-charged backbone that binds to nucleic acids. To increase the solubility and reduce aggregation, Polyethylene glycol (PEG) can be added in. Additionally, to enable the endosomal escape of this carrier, the DMMAnMel-shielded melittin (CPPs) can also be added in [63].

CPPs conjugated polymer enhances the cellular uptake of the molecular cargos, including the chemotherapy drug doxorubicin and the model molecule coumarin, when compared to the polymer without CPPs conjugation [64,65,66,67]. Cellular uptake efficiency of 5(6)-carboxy fluoresce transferred with the R9-polymer is increased 25 times when compared with that of the polymer [68]. *In vitro* data demonstrated that TAT modified cystamine bisacrylamide-diaminohexane (CBA-DAH) polymer enhanced cellular uptake [67]. In addition, TAT-poly-lysine [69] and the oligoarginine-linked polymer also enhance the transfection efficacy [70]. Similarly, TAT-conjugated methoxy poly (ethylene glycol) (MPEG)/poly (epsilon-caprolactone) (PCL) copolymers with disulfide linkage [71], showed a high-efficiency transfection and low cytotoxicity when the nanoparticles range from 100 to 200 nm encapsulated plasmid DNA or siRNA.

Many polymeric nanoparticles are designed for efficient delivery, and the CPPs-modified particles showed a trend of increased particle size and a looser complex [72]. However, TAT-modified nanoparticles exhibited the reversed result, as the polymer size decreased after modifying with TAT [73]. In conclusion, the crosslink between CPPs and the polymer is the key parameter for efficient CPPs modification; the valid linker MPEG-PCL disulfide couplings between TAT and polymer can significantly improve the delivery efficiency [74].

## 4. Combination of CPPs with Cationic Peptide

The cationic peptide could intensively bind to the cargos with negative charge such as nucleic acids. When CPPs are conjugated to cationic peptides in a covalent binding manner, they can deliver the cationic peptide with desired cargos to certain cells or tissues. The CPPs are also short stranded cationic peptides. TAT, a branch of CPPs, has been developed as a vector to deliver DNA and resulted in a four- to eight-fold higher gene expression when compared to Polyethylenimine (PEI), another popular transfection reagent [75].

Results other than those mentioned above could be attributed to the structure of the CPPs-peptide complex. Positive charge on the CPPs binds to the negative charge of nucleic acids, a behavior that is an important factor for nucleic acid delivery. A siRNA delivery experiment showed that CPPs with cationic peptides for siRNA delivery was significantly dependent on the types of CPPs and the length of the cationic peptides [76]. The experiment reported that the siRNA was bound to the MAP (model amphipathic peptide) based 21-mer oligolysine (K21-PDP), and the R6 or MAP CPPs was conjugated to the complex of siRNA-K21-PDP. The results showed that MAP based siRNA delivery efficiency was 170 and 600 folds greater at 1 h and 6 h post-transfection, respectively, when compared to the CPPs-based R6-K21-PDP vector [77]. As a result, the MAP-attached complex induced the comparable GFP silencing effects with the GFP siRNA transferred by the Lipofectamine 2000, which is a commercial siRNA delivery vector [77]. The consequence of this binding between nucleic acids and CPPs-modified cationic peptide led to the neutralization of positively charged CPPs, and the reduced or abrogated internalization efficiency of CPPs.

To solve the issue above, a new delivery method of nucleic acids emerged with the CPPs-modified dsRNA (double strand RNA) binding domain fusion protein (PTD-DRBD), which binds to the siRNA with a higher avidity [37,78,79], and therefore resulting in the PTD-mediated cellular uptake. The PTD-DRBD could deliver siRNA and induce the rapid RNA interference with high efficiency in various cells (e.g., the primary T cell, human umbilical vein endothelial cells, and human embryonic stem cells, *etc.*) with no cytotoxicity both *in vitro* and *in vivo*. The CPPs of TAT conjugated with U1A dsRBD as siRNA carrier (TAT-U1A dsRBD) [80] can also transfer siRNA into cells, but all the molecular complex of TAT-U1A dsRBD with siRNA are localized mainly in the endosome instead of cytoplasm.

**Figure 3:**
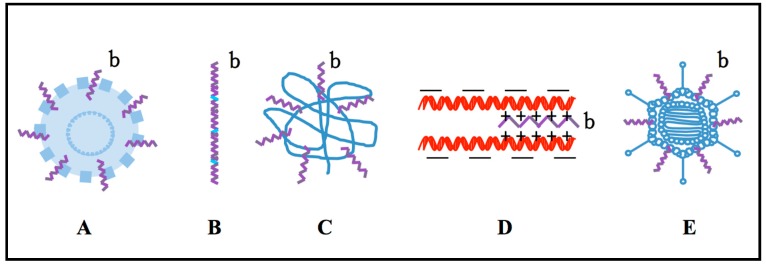
CPPs-based drug carriers. (**A**) Nanoparticles modified with CPPs; (**B**) poly CPPs as the vector; (**C**) The CPPs-loaded nanoparticles; (**D**) Nanoparticle formed by the bounding of CPPs with positive charge to drugs and cargoes with the negative charge; (**E**) Nanoparticles formed when CPP coated the carriers. b represents CPPs in the figure. b, colored in purple in above figure panel stands for CPPs; blue color represents nanoparticle in panel **A**, chemical links in panel **B**, Drugs in panel **C**, negative charge drugs (e.g., siRNA, *etc.*) in panel **D**, and other carriers (e.g., viral vectors, *etc.*) in panel **E**.

Nanoparticles with a diameter under 100 nm have been employed in various drug deliveries and have emerged as another carrier. Like other delivery carriers, they also have some restrictions that need to be overcome for further utilization. These issues are summarized as side effects including low efficiency and crossing the BBB. To improve the overall performance and effectiveness of nanoparticles in the field of drug delivery, several approaches have been developed, including the surface-modification of nanoparticles by aggregating with CPPs. Meanwhile, the positive charges of the CPPs can bind to the negative charges of drug, siRNA, DNA, and shRNA; and also can be coated with virus to form nanoparticles (Figure 3) [81,82,83].

Nanoparticles attached with CPPs improve the efficiency of drug delivery when compared to the non-modified CPPs-based nanoparticles [84,85]. For example, the LMWP (CPPs of low molecular weight protamine)-nanoparticles exhibited significant increased cellular accumulation when compared to the unmodified nanoparticles [86]. Gold nanoparticles modified with CPPs also enhance the cellular uptake [87]. In addition, the magnetic nanoparticles (MNPs) coated with either polyArg, polylysine (pLys) and PEI were evaluated for siRNA delivery; consequently, gene silencing was obtained from a brain cancer cell line C6, breast cancer cell line MCF7, and prostate cancer cell line TC2. However, it was reported that nanoparticle-pArg-siRNA can deliver a remarkably higher quantity of siRNA than that of nanoparticle-pLys-siRNA, furthermore, nanoparticle-PEI-siRNA comes with a higher cell viability when doing transfection [88]. Similarly, another study showed that the target gene expression could be increased 100 fold when using CPPs-based nanoparticles for DNA delivery as compared to the mutant TAT2-M1 that has no activity; and it was still four to eight times higher when compared to PEI, a popular cationic polymer for transfection [75].

In addition to the enhancement of cellular uptake, the CPPs modification can also improve the endosomes escape [89]. Since CPPs have been reported in a large scope to improve endosomal escape [47], nanoparticles in cooperation with octaarginine provide an ideal carrier system for delivering siRNA to the cells, and consequently induce a specific knock-down of targeted genes [90,91]. Thus, RNA interference is a promising strategy for gene therapy.

The number of CPPs on the nanoparticle surface is a pivotal property for efficient delivery in surface-modified nanoparticles. The uptake of nanoparticles is nonlinearly in accordance with the increase of CPPs amounts on the nanoparticle surface, and the delivery efficiency is 100-fold higher at the condition of 15 CPP (TAT) per cross-linked iron oxide (CLIO) particle [92]. In addition, the release rate from cells is also dependent on the amount of CPPs loading [93]. Meanwhile, the preparation methods of CPPs-based nanoparticles also affect the overall efficiency of drug delivery. It is evident that the nanoparticles FA-PC/R8-PC/pDNA-M and FA-PC/R8-PC/pDNA-L undergoing different preparation processes with an addition of CPPs formed different structures of nanoparticles, and thus showing different delivery efficiencies [94].

Another interesting application of nanoparticles conjugated with CPPs is that it can cross BBB and deliver drug components, while nanoparticles without TAT are unable to cross the BBB [84]. Nanoparticle size also plays an important role in its degree of permeability. For example, PEGylated gold nanoparticles can be located in the nucleus when the particle size is 2.4 nm as opposed to delivered the cytoplasm and the membrane when the particle size is 5.5 and 8.2 nm, respectively. Meanwhile, the nanoparticles cannot enter into the cells and are located at the cellular periphery when the particle size is 16 nm or larger [95]. In conclusion, the smaller the nanoparticle size, the closer to the nucleus it travels.

## 5. Binding of CPPs with the Viral Carrier

Adenovirus (Ad) is one of the viral vectors most widely used for gene delivery; they have been widely used in gene therapy studies due to their high transduction efficiency [96,97]. However, their serious shortcomings including immunogenicity, promiscuous tropism, and the inability to efficiently infect certain types of cells limited their translational utility. Briefly, Ad vectors based delivery is dependent on the cell surface receptor such as the coxsackievirus and adenovirus receptor (CAR), and has difficult delivering genes to the cells that lack CAR expression [98,99].

To improve the ability of an Ad-based vector to efficiently transform cells that lack the CAR, the peptide TAT-G-C [100] was chemically conjugated to the Ad vector with the cross linker 6-maleimidohexanoic acid N-hydroxysuccinimide ester. The conjugation of Ad vector demonstrated that it has a significantly enhanced efficiency of gene delivery in B16BL6 cells when compared to the wild type adenovirus vector. A similar experiment was also applied to the TAT related peptide. When G-R5-Q-R3-P2-Q-G-C was bound to the adenovirus vector, it showed an enhanced efficiency of the gene delivery in the B16BL6 cells, 50-fold greater than that of the wild type adenovirus vector [101]. Recently, the Ramsey group produced the vector by using PEGylating Ad, which packages a lacZ reporter gene, and then conjugating CPPs to form CPP-PEG-Ad particles. Meanwhile, these studies compared the effectiveness of four different CPPs (Pen, Tat, Pep1, and pArg). The results showed that CPP-PEG-Ad particles transduced the lack of CAR cells significantly better than unmodified Ad; Pen, the most effective CPP, produced an 80-fold improvement in transduction compared to the unmodified virus [102].

The CPPs can also be gene recombined into the adenovirus and produce the CPPs-modified Ad vectors. The gene recombined Ad vectors AVV- TAT (HI)-L2 and AVV-TAT(C)-L2 including a TAT improved the gene delivery efficiency at approximately 50 to 500 folds higher gene expression when compared to the wild type vector [103]. Finally, the recombined Ad vectors with CPPs dramatically increased gene transduction efficiency in the lack of CAR cells when compared to the wild type vector [104]. Addition of CPPs to virus vector can deliver genes to the cells that lack CAR expression.

## 6. Calcium and CPPs

Calcium is an important factor for cell signal transport and can enhance cellular uptake of CPPs as the cargo carrier [105,106,107]. For instance, the HeLa cellular uptake of TAT increased 44 folds when 6 mM Ca^2+^ was added into the culture medium [106], the CPPs dimer form with siRNA can be condensed, and consequently improve the cellular uptake of siRNA delivery [108,109,110].

Although the mechanism of Ca^2+^ as a delivery enhancer has not yet been clearly proven [111,112], Ca^2+^ is mainly involved in endocytosis [111], and the disruption of endosomes [113,114] improves the transfection and formation of nanoparticles with calcium [108,115]. Notably, the CPPs-based drug transferring induced the influx calcium [18,116]. Upon the addition of calcium, the “loose” complexes of siRNA/CPP can be condensed into small nanoparticles and thus, offers high efficiency and low toxicity gene silencing.

## 7. Conclusions

**Figure 4:**
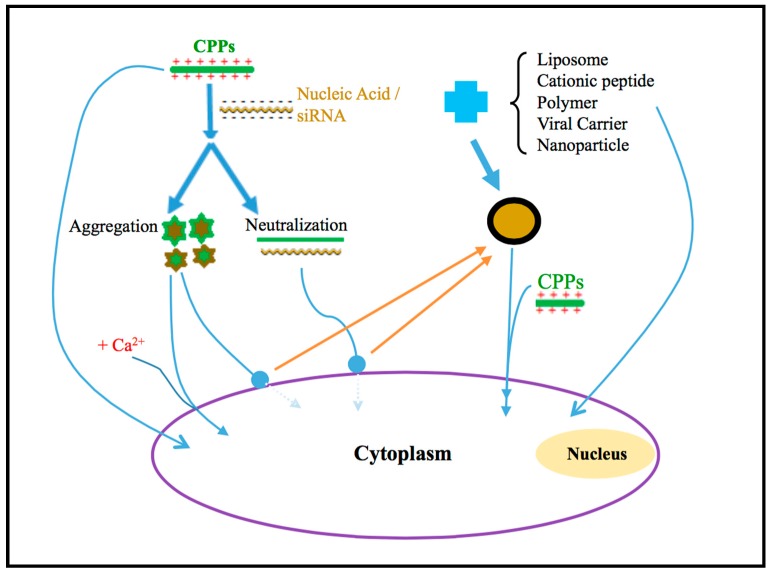
The potential use of CPPs. CPPs has been widely used as modulators or vehicles for the delivery of various biological products into cells. In order to overcome the neutralization and aggregation of CPPs that result in the inability to cross the cell membrane, calcium can be used to improve the nucleic acid delivery. The nucleic acids could be assembled with the typical drug carrier (e.g., liposome, cationic peptide, polymer, viral carrier and nanoparticle), and then be further modified with CPPs, consequently dramatically enhancing the nucleic acid transferring efficiency.

The application of CPPs is one of the most attractive approaches for drug delivery, as summarized in Figure 4. The cellular uptake of the carrier system, including liposome, polymer, cationic peptide, nanoparticles, and AVV are dramatically enhanced without significant cytotoxicity after modifying with CPPs. Moreover, the calcium can be further applied to enhance the cellular uptake of CPPs.

## Abbreviations

**Abbreviation**	**Full Name**
Arg	Arginine
Asp	Aspartic acid
AVV	Adeno-associated virus
BBB	Blood brain barrier
C	Cysteine
CAR	Coxsackievirus and adenovirus receptor
CBA-DAH	Cystamine bisacrylamide-diaminohexane
CNS	Central nervous system diseases
CPP	Cell penetrating peptide
dsRBD/DRBD	Doubled RNA binding domain
G	Glycine
Glu	Glutamic acid
H	Histidine
HA2	hemagglutinin peptide
HIV	Human immunodeficiency virus
L	Leucine
LMWP	Cell penetrating peptide of LMWP
Lys/K	Lysine
MAP	Model amphipathic peptide
MNPs	Magnetic nanoparticles
MPEG	Methoxy poly (ethylene glycol)
P	Proline
PCL	Poly(epsilon-caprolactone)
PEG	Polyethylenglycol
PLL	Poly-L-Lysine
pLys	Polylysine
PTD	Protein transduction domain
PTD-DRBD	CPP of PTD modified dsRNA binding domain
Q	Glutamine
R	Arginine
R8	Polyarginine8
RNAi	RNA interference
shRNA	Short hairpin RNA
siRNA	Small interfering RNA
TAT	Cell penetrating peptide of TAT
TAT-PEG-MPLs	TAT-conjugated PEGlated Magnetic polymeric liposome
TAT-U1A dsRBD	TAT conjugated with U1A dsRBD
TP 10	Transportan10
U1A	U1 small nuclear ribonucleoprotein A
V	Valine
